# A Case in Office Practice

**Published:** 1888-06

**Authors:** J. W. Jay


ARTICLE II.

### A CASE IN OFFICE PRACTICE.

BY J. W. JAY, M. D., D. D. S.

On the tenth of last November a lady came into my office and asked me if I could extract a tooth for her. I told her I thought I could. She then informed me that Dr. Kelsey, one of our prominent young physicians, would be in presently to give her ether. He soon came, and she took her seat in my chair. In appearance she was a lady in possession of rather more than ordinary health, and I should think would weigh about 130 pounds. Placing her in the chair at about an angle of 45 degrees, and supposing that the Dr. had made all the necessary examinations in regard to her health at their previous interview, in regard to her taking ether, I asked her no questions. On examining her mouth, I found there were two teeth to remove instead of one, and told the doctor that I was ready. He immediately began the administration of the ether, and in just three minutes from the first inhalation, the teeth were out, taking them while she was under the primary effects of the ether. Still retaining my position by her side, and Dr. Kelsey standing in front of the chair, both of us expecting, of course, that in a few minutes she would open her eyes and show symptoms of returning consciousness, but in this we were doomed to disappointment. Up to this point her breathing was natural and pulse normal.

Very soon, however, to our dismay, her breathing grew shorter and shorter, and then ceased altogether. I sprang to her, and began the process of artificial respiration, by standing behind the chair, and a little above her, and placing my hands, one under each breast, at the margin of the short rlbs, and pressing upward, and then relaxing the pressure alternately. Keeping up this process for a few seconds without any appreciable benefit whatever, the doctor hurriedly suggested that we lay her on the floor, and we lost no time in complying with his suggestion. As soon as she was in this position, the doctor preceeded with the artificial respiration without any apparent benefit.

Her eyes were closed from the start, and she was as limp as a rag, and her countenance, by this time, had assumed the livid hue of death.

We saw at once that something must be done and done quickly. So I gathered her by the legs and lifted her up, her head hanging down, and her legs resting one on each of my shoulders. All this time she had not breathed once, and I distinctly remember of wondering to myself, how it could be possible for anyone to be so long without drawing a single breath and live, for it seemed to me an age, therefore, I said to the doctor it is no use, she is dead.

He placed his ear to her chest, and said her heart still beats, then said I, we will work as long as the heart continues to act. At this critical moment, she showed manifestations of returning life, by a feeble respiration, accompanied with a suppressed groan, and at a long interval another, and then another. I held her in the inverted position until her breathing seemed to be thoroughly established, which produced a sense of relief to us both, more easily imagined then described. We placed her again in a horizontal position on the floor, and, of course, thought that now she surely was all right. The doctor exclaimed, "My God, that is the closest call I ever had." But how soon were our cherished hopes frustrated.

In a few seconds, after we had laid her down, we had a repetition of the same condition. I took her again by the legs, and held her in an inverted position as before. This time we were sure that she had gone to the "bourne whence no traveler ever returns." But as good luck, combined with our efforts, the doctor doing what he could at artificial respiration, fortune once more smiled upon us, and she breathed again, with a groan, and a second time we breathed easier, with the hope, that surely, this is a permanent return, and we again laid her on the floor. But how were we startled soon after, to see her relapse into a state of stupor, apparently more profound than before. This time the doctor laid her breast bare, by taking hold of her collar and dress, under her chin, making the buttons fly in every direction wtth a zip.

He gave her hypodermic injections of whiskey, and applied cloths wrung out of cold water to her breast, without effect. She laid there seemingly as lifeless as a corpse. By this time I felt considerably played out. A strong man from the country, who had come in to have some work done, was sitting by, looking on with intense interest. I asked him to take her up, as I had done, to which he readily consented, and held her in an inverted position as before, until her breathing was restored, when she was again placed upon the floor. Suffice it to say that the inverting process was resorted to the sixth time before continued respiration was restored.

When this was accomplished, we laid her on a lounge, and her respiration being quite feeble, the doctor gave her two or three more hypodermic injections of whisky. The heart's action also being very feeble, the doctor sent out and obtained some nitrate of amyl, and had her inhale a portion, with very happy effect.

During the whole time we kept watch of her tongue, and saw that it did not drop down her throat and rest on the epiglottis, and thereby produce strangulation.

It was at least twenty minutes after she began breath ing regularly, before she showed the least evidence of returning consciousness. Observing the clock, I discovered that it had been about an hour since the doctor began the administration of the ether.

Nothing further occurred in reference to the case to mar our expectations of a speedy recovery of our patient, and right glad were we, when we felt assured that a restoration was at hand.

She commenced taking the ether at a few minutes after 2 p. m., and a little after 5 p, m., she walked home, a distance of five or six squares.

Squib's ether was used, said by surgeons to be the best in the world, and less than three ounces were given by actual weight, and from a can the doctor opened for the first time in the office, in my presence.

An exceedingly interesting account of a case, called "Sims' Case," related by J. Marion Sims himself, in a paper read before the British Medical Association, of the syncope and resuscitation of the patient. He was operating upon a French Countess—young, beautiful, accomplished—for vesico-vaginal fistula, the result of her first accouchement. The account is extracted from his paper, and given in vol. 3, page 125 of American System of Dentistry, by Dr. Litch, in his able and exhaustive paper on Anæsthesia and Anæsthetics, which will richly repay any man to read.

In Sims' Case, chloroform was used instead of ether. But to all appearance, the syncope; as the result of anæsthesia, was the same; and in the treatment of our patient, we endeavored to follow the same course adopted in "Sims' Case." As the great Nelaton said, who was standing by watching and giving orders on that occasion "certainly, there is nothing else to do."

About two weeks previous to our experience with this lady, Dr. Bond, one of the prominent physicians of this city, had a similar experience with her in the office of Dr. Hamilton, one of my neighbor dentists. This time she had but one tooth extracted, After an hour or more of vigorous effort on the part of the two doctors, she came out all right. When she was fully restored, the doctor very emphatically endeavored to impress her with the fact, that she was not an acceptable subject for the action of ether, and for her never to attempt to take it again under any circumstances whatever, reminding her, that if she did, in all human probability, she would wake up in another state of existence.

She gave neither Dr. Kelsey or me the least intimation of the advice and caution that Dr. Bond had given her in reference to taking ether again. If she had, she would have met with a prompt refusal.—*Dental Register.*

